# Golden Lady

**DOI:** 10.5826/dpc.1101a134

**Published:** 2021-01-29

**Authors:** Andrina Neff, Alexandra Valeska Matter, Isabel Kolm

**Affiliations:** 1 Department of Dermatology, University Hospital of Zurich, Switzerland

**Keywords:** plane xanthoma, monoclonal gammopathy, lymphoproliferative disease, hyperlipidemia

## Case Presentation

**Figure 1:**
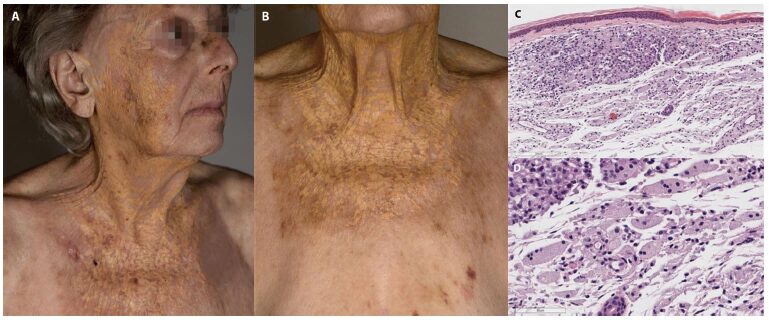
(A, B) Remarkable presentation of plane xanthoma on the neck and lateral face with a golden hue in a 93-year-old woman. (C, D) Histopathology of plane xanthoma with CD68-positive foamy macrophages in the papillary dermis.

A 93-year-old woman presented with confluent yellow plaques with an intense golden glow symmetrically on the neck and lateral face (Figure 1, A and B) mimicking a golden camouflage. The histopathology showed CD68-positive foamy macrophages in the papillary dermis (Figure 1, C and D), leading to the diagnosis of plane xanthoma. Our patient showed mild dyslipidemia (total cholesterol 6.4 mmol/l [<5.0 mmol/l]). Clinical stigmata for familial hyperlipidemia were not present. Due to the distribution pattern, underlying hematological disorders were ruled out.

## Teaching Point

The location of plane xanthomas can serve as a clue to a particular underlying disease. Plane xanthomas in a normolipemic patient with favored distribution on the neck, upper trunk, and flexural folds should prompt a search of underlying hematological disorders such as monoclonal gammopathy or lymphoproliferative disease [1,2]. Plane xanthomas can precede such disorders by several years; therefore, a regular follow-up is recommended.
